# The availability of neither D2 nor CP43 limits the biogenesis of photosystem II in tobacco

**DOI:** 10.1093/plphys/kiaa052

**Published:** 2020-12-09

**Authors:** Han-Yi Fu, Rabea Ghandour, Stephanie Ruf, Reimo Zoschke, Ralph Bock, Mark Aurel Schöttler

**Affiliations:** Max-Planck-Institut für Molekulare Pflanzenphysiologie, Am Mühlenberg 1, D-14476 Potsdam, Germany

## Abstract

The pathway of photosystem II (PSII) assembly is well understood, and multiple auxiliary proteins supporting it have been identified, but little is known about rate-limiting steps controlling PSII biogenesis. In the cyanobacterium *Synechocystis* PCC6803 and the green alga *Chlamydomonas reinhardtii*, indications exist that the biosynthesis of the chloroplast-encoded D2 reaction center subunit (PsbD) limits PSII accumulation. To determine the importance of D2 synthesis for PSII accumulation in vascular plants and elucidate the contributions of transcriptional and translational regulation, we modified the 5′-untranslated region of *psbD* via chloroplast transformation in tobacco (*Nicotiana tabacum*). A drastic reduction in *psbD* mRNA abundance resulted in a strong decrease in PSII content, impaired photosynthetic electron transport, and retarded growth under autotrophic conditions. Overexpression of the *psbD* mRNA also increased transcript abundance of *psbC* (the CP43 inner antenna protein), which is co-transcribed with *psbD*. Because translation efficiency remained unaltered, translation output of *pbsD* and *psbC* increased with mRNA abundance*.* However, this did not result in increased PSII accumulation. The introduction of point mutations into the Shine–Dalgarno-like sequence or start codon of *psbD* decreased translation efficiency without causing pronounced effects on PSII accumulation and function. These data show that neither transcription nor translation of *psbD* and *psbC* are rate-limiting for PSII biogenesis in vascular plants and that PSII assembly and accumulation in tobacco are controlled by different mechanisms than in cyanobacteria or in *C. reinhardtii*.

## Teaser

The mechanisms controlling photosystem II accumulation in vascular plants differ from those in cyanobacteria and the green alga *Chlamydomonas reinhardtii*.

## Introduction

Photosystem II (PSII), the water-plastoquinone oxidoreductase protein supercomplex of oxygenic photosynthesis, catalyzes the first step of linear electron flux in the thylakoid membranes of cyanobacteria and photosynthetic eukaryotes (Shen, 2015). Electron transfer within PSII is initiated by a light-induced charge separation at the reaction center (RC) chlorophyll-a dimer P_680_, which then transfers one electron to the first quinone acceptor Q_A_. From Q_A_, the electron is transferred to the plastoquinone-binding site (Q_B_-site) and reduces plastoquinone to plastosemiquinone. Following a second charge separation, the subsequent reduction of the plastosemiquinone to plastoquinol is coupled to the uptake of two protons from the stroma. Plastoquinol is released into the thylakoid membrane and re-oxidized at the cytochrome b_6_f complex (cyt b_6_f). After each charge separation, P_680_^+^ is reduced by the oxygen-evolving Mn_4_O_5_Ca complex (OEC) near the luminal surface of PSII. After four oxidation steps, two water molecules are oxidized to molecular oxygen, and the Mn_4_O_5_Ca cluster is reduced again by the four electrons abstracted from the two water molecules (Dau et al., 2012; Vinyard and Brudvig, 2017).

PSII functions as a dimer, and each monomer is composed of more than 20 core subunits and additional peripheral light-harvesting complex (LHC) antenna proteins. With a molecular mass of up to 1,300 kDa, the PSII–LHC supercomplexes are the largest complexes of the photosynthetic apparatus (Dekker and Boekema, 2005; Kouřil et al., 2012; Shen, 2015). The PSII RC core is formed by the D1 and D2 heterodimer that binds all redox-active cofactors necessary for rapid electron transfer from water to plastoquinone. D1 and D2 are encoded in the chloroplast genome (plastome) by the *psbA* and *psbD* genes, respectively. An additional redox-active cofactor, the heme of cytochrome b_559_ (cyt b_559_), is bound by PsbE and PsbF, which are also plastome-encoded. Cyt b_559_ is an essential structural component and required for PSII assembly (Pakrasi et al., 1989; Morais et al., 1998; Swiatek et al., 2003), but its physiological function is enigmatic. It has been suggested to mediate a cyclic electron flux within PSII when the PSII donor side is inactive (Shinopoulos and Brudvig, 2012; Takagi et al., 2019) and may function as a plastoquinol oxidase (Bondarava et al., 2003; Bondarava et al., 2010).

The PSII RC is surrounded by the inner antenna proteins CP47 (PsbB) and CP43 (PsbC), also encoded in the plastome, and multiple membrane-intrinsic low-molecular mass subunits encoded either in the plastome or the nucleus. Some of these subunits are essential for PSII accumulation or function, while others are not (reviewed by Shi et al., 2012; Plöchinger et al., 2016). Additionally, the three nuclear-encoded extrinsic subunits PSBO, PSBP, and PSBQ are associated with the luminal side of PSII and stabilize the OEC (Bricker et al., 2012).

The process of de novo PSII assembly is largely conserved from cyanobacteria to vascular plants, except that the subcellular localization of some steps varies (Komenda et al., 2012; Nickelsen and Rengstl, 2013). Assembly proceeds in a modular fashion and starts with cyt b_559_, which stably accumulates even in the absence of the other PSII RC subunits (Müller and Eichacker, 1999; Kanervo et al., 2008; Plöscher et al., 2009; Schmitz et al., 2012). Subsequently, D2 is co-translationally inserted into the thylakoid membrane (Zoschke and Barkan, 2015) and binds to cyt b_559_, forming the D2-cyt b_559_ subcomplex (Komenda et al., 2004). The addition of a complex consisting of pre-D1, a D1 protein precursor with a short C-terminal extension, and PsbI leads to the formation of the “RC-like complex” (Dobakova et al., 2007), which is stabilized by multiple auxiliary proteins (Li et al., 2019). After maturation of the D1 protein by the luminal C-terminal processing protease CTPA (Che et al., 2013), the “RC47 subcomplex” is formed by binding of CP47 (PsbB; Komenda et al., 2004) and the rapid addition of PsbH, PsbR, and PsbTc (Rokka et al., 2005). Finally, CP43 (PsbC), PsbK, and PsbZ bind to the RC (Rokka et al., 2005; Boehm et al., 2011). This complex is photoactivated by stepwise assembling the Mn_4_O_5_Ca cluster, which is further stabilized by binding the three luminal subunits (Mamedov et al., 2000; Mamedov et al., 2008). Ultimately, two PSII monomers form a PSII dimer and bind additional LHCs (Shevela et al., 2016). During the assembly process, more than 20 auxiliary proteins located in the stroma, the thylakoids, and the lumen transiently bind to PSII. They protect and stabilize all assembly intermediates except for the early D2-cyt b_559_ subcomplex, which appears to accumulate without the support of auxiliary proteins (Shi et al., 2012; Nickelsen and Rengstl, 2013; Plöchinger et al., 2016). Some auxiliary proteins mediate chlorophyll and cofactor insertion into the RC and inner antenna proteins of the nascent complex (Bučinská et al., 2018; Hey and Grimm, 2020).

In vascular plants, PSII contents are highly variable (reviewed by Schöttler and Tóth, 2014). In Arabidopsis (*Arabidopsis thaliana*), PSII contents increase almost four-fold from 2.5 to 9 mmol PSII per mol chlorophyll, at the expense of the peripheral LHCII, when the actinic light intensity increases from 35 to 600 µE m^−2^ s^−1^ (Bailey et al., 2001). Similar changes have been observed in tobacco (*Nicotiana tabacum*; Petersen et al., 2011; Schöttler et al., 2017) and in *Chamerion angustifolium* (Murchie and Horton, 1998). The mechanisms, by which these adjustments are achieved, are largely unknown.

In principle, PSII accumulation could be limited by the biosynthesis of one of its subunits, by the synthesis and insertion of redox cofactors and chromophores, especially chlorophyll and carotenoids into the nascent complex, or via changes in the abundance of auxiliary proteins. Because PSII assembly starts with cyt b_559_, a limiting role of cyt b_559_ synthesis for the entire assembly process is a reasonable assumption. Supporting this scenario, in the cyanobacterium *Synechocystis* PCC6803, deletion of the *psbEFLJ* operon comprising the subunits of cyt b_559_ abolishes accumulation of both D1 and D2 (Pakrasi et al., 1989). Radiolabeling studies reveal that while the absence of D1 in the mutant is mainly attributable to rapid turnover of the unassembled protein, the presence of cyt b_559_ is a prerequisite for translation of D2 (Komenda et al., 2004). However, because a fraction of cyt b_559_ forms part of a higher molecular mass complex of unknown function that does not comprise other PSII subunits, cyt b_559_ cannot be the only factor controlling D2 synthesis. Because D2 accumulates in a Δ*psbA* mutant, the downstream assembly partner D1 does not control *psbD* translation (Komenda et al., 2004). Interestingly, in all of these mutants, the inner antenna proteins CP43 and CP47 accumulate, indicating that their synthesis is independent of the presence of those RC subcomplexes, into which they later assemble (Pakrasi et al., 1989; Komenda et al., 2004). CP47 forms a pre-assembled pigment–protein complex together with PsbH, PsbL, and PsbT, and CP43 associates with PsbK (Boehm et al., 2011). Clearly, not all subunits of PSII are subject to a tight control of their synthesis by the presence of the essential RC core subunits. However, all observations in *Synechocystis* PCC6803 point to a limiting role of cyt b_559_ and other, so far unknown factors for the synthesis of D2 and thereby of functional PSII.

Data obtained in photosynthetic eukaryotes suggest major differences in the regulation of PSII biogenesis: In the green alga *Chlamydomonas reinhardtii*, accumulation of cyt b_559_ is a prerequisite for PSII assembly (Morais et al., 1998). However, different to *Synechocystis*, cyt b_559_ does not control the synthesis of D2, as in the absence of cyt b_559_ in a Δ*psbE* mutant, up to 5% of wild-type (WT) levels of D2 still accumulated, while D1 was not detectable anymore (Morais et al., 1998). Instead, in *C. reinhardtii*, already relatively small changes in *psbD* mRNA accumulation and translation have a strong influence on PSII accumulation, supporting a limiting function of D2 synthesis for PSII accumulation. A point mutation in the *psbD* promoter reduces both *psbD* mRNA accumulation and D2 protein content to 35% of the WT level (Klinkert et al., 2005). Furthermore, because the AUG start codon is part of an mRNA secondary structure, translation initiation of *psbD* is controlled by the translational activator RBP40 and the RNA stabilization factor Nac2, which open up the mRNA secondary structure and thereby facilitate translation initiation (Schwarz et al., 2007). Mutations of this repressor element not only result in increased translation of the *psbD* mRNA, but also lead to a 20% increase in PSII accumulation, clearly supporting a limiting role of PsbD in PSII biogenesis (Klinkert et al., 2006).

In vascular plants, similar to *C. reinhardtii*, a limiting function of cyt b_559_ can likely be excluded. While knock-out of *psbE* or *psbF* abolishes the accumulation of both D1 and D2 (Swiatek et al., 2003), free cyt b_559_ accumulates not only in etioplasts (Müller and Eichacker, 1999; Kanervo et al., 2008), but also in chloroplasts with severely disturbed sugar-phosphate metabolism and redox poise (Schmitz et al., 2012). Assuming that, similar to the situation in *C. reinhardtii*, D2 plays a limiting role, the underlying molecular mechanisms likely differ, because neither RBP40 nor Nac2 have orthologs in vascular plants. Also, while *psbD* is encoded by a monocistronic mRNA in *C. reinhardtii*, in vascular plant plastomes, it forms an operon with *psbC* (Yao et al., 1989; Christopher et al., 1992; Adachi et al., 2012). In tobacco, the main promoter of this operon is located 905-bp upstream from the ATG codon of *psbD*. Additionally, a promoter located 194-bp upstream from the start codon of *psbC* is located within the protein-coding region of *psbD* (Yao et al., 1989). Because the reading frames of *psbD* and *psbC* overlap by 17 nucleotides in tobacco, it has been suggested that their translation is partly coupled. At least some of the ribosomes released after termination of *psbD* translation may immediately rebind to the *psbC* 5′-untranslated region (UTR) and initiate CP43 synthesis (Adachi et al., 2012). However, because *psbC* can be also expressed as a monocistronic transcript (Yao et al., 1989), translational coupling is not a prerequisite for PsbC synthesis.

While in vascular plant chloroplasts, translation regulation is usually considered to be more important than transcript abundance (reviewed by Zoschke and Bock, 2018), a limitation of photosynthetic complex biogenesis by transcript abundance was recently reported for the PetA subunit of the cyt b_6_f in tobacco (Schöttler et al., 2017). To determine if D2 or CP43 plays a limiting role for PSII accumulation in vascular plants, here we have generated transplastomic tobacco mutants with altered *psbD–psbC* transcript abundances. To assess a potential role of *psbD* translation regulation, we altered *psbD* translation initiation by the introduction of point mutations into the ATG start codon. Similar approaches have been used by Rott et al. (2011) to reduce *atpB* translation, and by Moreno et al. (2017) to repress translation of ClpP, the chloroplast-encoded catalytic subunit of the stromal Clp protease (Nishimura and van Wijk, 2015). We also mutated the Shine–Dalgarno (SD)-like sequence in the 5′-UTR of *psbD*. The SD interacts by complementary base pairing with the anti-SD sequence in the 16S ribosomal RNA to ensure proper positioning of the translation initiation complex close to the start codon. In a transplastomic tobacco line carrying a mutation in the anti-SD sequence, *psbD* ranked among the genes whose translation was most severely affected (Scharff et al., 2017). Our data suggest that *psbD* transcript abundance is critical in controlling D2 synthesis, but D2 is not the rate-limiting subunit for the accumulation of PSII in tobacco. We also show that the regulation of PSII biogenesis is markedly different between cyanobacteria, *C. reinhardtii* and vascular plants.

## Results

### Generation of transplastomic tobacco lines with altered *psbD* expression

**Figure 1:**
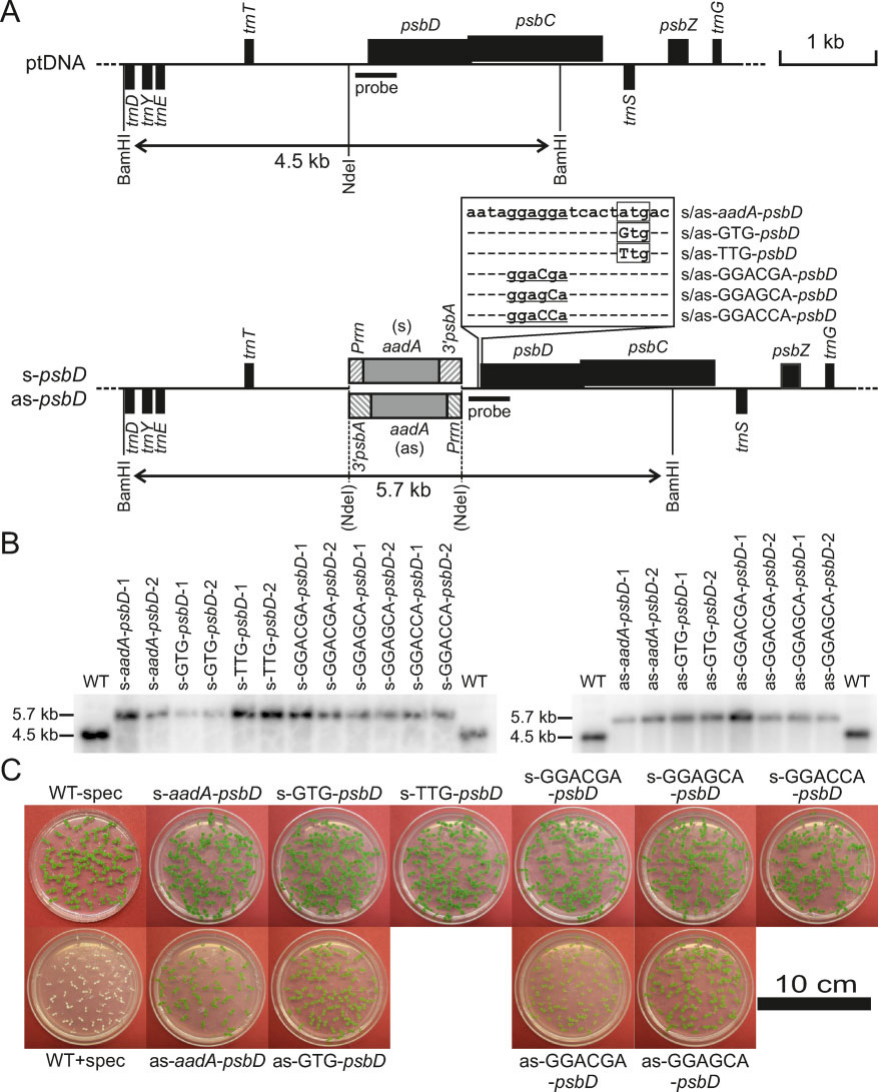
Generation and molecular characterization of *psbD* mutants. (**A**) Physical maps of the WT and mutant plastomes including all restriction sites used for cloning and RFLP analyses. Genes above the line are transcribed from left to right, genes below the line are transcribed in the opposite direction. The position of the probe used for RFLP analyses and the sizes of the detected BamHI restriction fragments are indicated. (**B**) RFLP analyses of transplastomic tobacco lines. DNA samples of transplastomic and WT plants were digested with the restriction enzyme BamHI (left panel: sense-mutants, right panel: as-mutants). (**C**) Inheritance tests to confirm homoplasmy. Seeds from the WT and the different sense and antisense mutants were germinated in the presence of spectinomycin (scale bar = 10 cm).

To dissect the contributions of transcriptional and translational regulation to the synthesis of the plastid-encoded D2 subunit, we generated chloroplast mutants with altered transcription of the *psbD–psbC* operon by disrupting the *psbD* 5′-UTR via insertion of the selectable marker gene *aadA* (for details, see the “Materials and methods” section). In one set of transplastomic mutants, the selectable marker, whose expression is driven by the strong *Prrn* promoter, was inserted in the plastome in sense orientation relative to *psbD*. These mutants will subsequently be referred to as “sense” mutants (s-mutants). Read-through transcription from the *aadA* gene is expected to result in strong over-expression of the downstream *psbD*–*psbC* transcription unit (Zhou et al., 2007; Lu et al., 2013; Zhang et al., 2015). In another set of mutants, the selectable marker was inserted in antisense orientation relative to *psbD*, so that no read-through transcripts can occur. These mutants are referred to as “antisense” (as)-mutants. Because insertion of the selectable marker in antisense orientation may negatively affect the expression of neighboring genes transcribed in the opposite direction (Loiacono et al., 2019), transcript accumulation of *psbD* could be strongly reduced in all as-mutants. Additionally, to alter translation initiation efficiency, we introduced point mutations into the translation initiation codon. The standard ATG translation initiation codon was replaced either by a GTG or TTG codon (Figure 1, A), which are both recognized by the chloroplast ribosome as translation initiation sites, but with lower efficiency than the ATG codon (Rott et al., 2011; Moreno et al., 2017). Furthermore, as Scharff et al. (2017) had shown the SD dependency of *psbD* translation, we mutated the SD of *psbD* from GGAGGA to GGACGA, GGAGCA, or GGACCA.

Transplastomic lines were obtained by particle-gun-mediated chloroplast transformation and selection for spectinomycin resistance (conferred by the *aadA* gene). All mutants could be generated and propagated in tissue culture under mixotrophic conditions. However, when plants were transferred to autotrophic growth conditions, neither the as-TTG-*psbD*-mutant plants nor the as-GGACCA-*psbD*-mutant plants survived. Both mutants could only be maintained in tissue culture, and consequently, did not produce seeds. Therefore, these mutants were excluded from further analysis. The homoplasmic state of all other mutants was tested by restriction fragment length polymorphism (RFLP) analysis using a *psbD*-specific probe and BamHI as restriction enzyme (as indicated in the map in Figure 1, A**)**. Two representative independent lines for each construct were analyzed (Figure 1, B). While the WT showed the expected restriction fragment of 4.5-kb size, all transplastomic mutants showed exclusively a larger fragment of 5.7 kb, which arises from the insertion of the *aadA* marker gene with its promoter and terminator. The presence of the point mutations was confirmed by DNA sequencing.

Absence of the smaller WT-like fragment in the mutants strongly suggested the homoplasmic state of all transplastomic lines. To ultimately confirm homoplasmy, we also performed seed germination tests on spectinomycin-containing medium. This is the most sensitive assay to distinguish between homoplasmic and heteroplasmic transformants (Svab and Maliga, 1993; Bock, 2001; Bock, 2015). As expected, cotyledons of WT seedlings germinated in the presence of spectinomycin were completely white, because inhibition of plastid translation prevents assembly of the photosynthetic apparatus (Figure 1, C). By contrast, all transplastomic lines produced a homogeneous population of green seedlings, thus unequivocally confirming their homoplasmic state. Interestingly, seedlings from the as-mutants were only pale green compared with those from s-mutants, which is in line with the chlorophyll-deficient phenotypes of as-mutants grown in soil (see below).

### Transcript accumulation from the *psbD* operon

**Figure 2:**
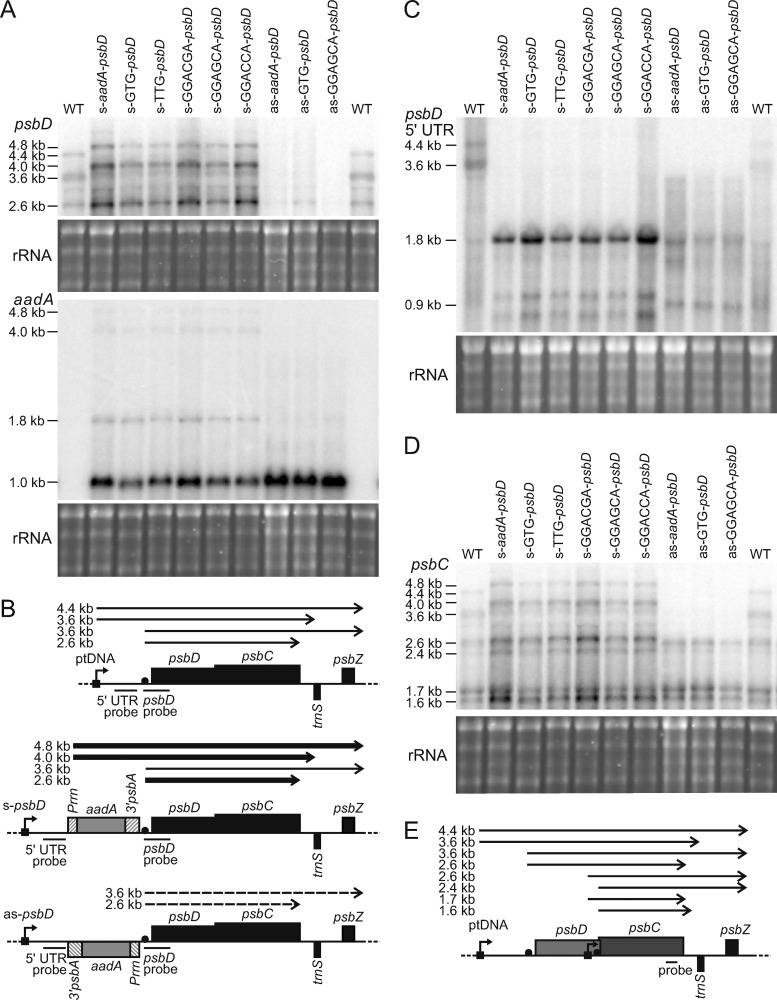
Changes in *psbD* and *psbC* transcript patterns and transcript abundance in transplastomic sense and antisense mutants. (**A**) Northern blots probed with *psbD*- (upper panel) and *aadA*-specific (lower panel) probes. As a loading control, the ethidium bromide-stained agarose gel prior to blotting is shown below each blot. (**B**) Schematic representation of the *psbD* operon and the transcript species giving rise to the bands observed in northern blots. (**C**) Northern blot hybridized to a probe against the 5′-UTR of *psbD*. (**D**) Probe against *psbC*. For this blot, the membrane used for the *aadA* blot in Figure 2, A was stripped and rehybridized. (**E**) Schematic representation of the *psbC*-containing transcripts detected by northern blot hybridization.

To determine general effects of the insertion of the selectable marker gene and the introduction of the point mutations on mRNA accumulation and processing, northern blot analysis was performed for all s-mutants, the as-*aadA–psbD* control line, and the as-GTG-*psbD* and as-GGAGCA-*psbD* mutants, which were viable under autotrophic conditions (Figure 2). Even though the as-GGACGA-*psbD* plants had produced some seeds (and, therefore, could be included in the germination assays; Figure 1, C), they only rarely survived autotrophically. Therefore, this mutant was excluded from further analyses. In the WT, using a *psbD*-specific probe, a complex pattern of transcripts between 4.4- and 2.6-kb size was observed. This pattern arises from mRNA cleavage at a processing site located at position −132 relative to the translation initiation codon (Adachi et al., 2012), and from transcriptional read-through into *psbZ*. The *psbZ* gene is encoded downstream of *psbC* on the same DNA strand (Figures 1, A and 2, B).

The mutants with *aadA* inserted in s-orientation revealed the presence of two novel bands, which represent read-through transcripts from the strong *Prrn* promoter in front of the *aadA* selectable marker gene through the *psbD*–*psbC* operon (transcript of approximately 4.0-kb size), or even through *psbZ* (approximately 4.8-kb size). Furthermore, the abundance of the processed dicistronic mature *psbD–psbC* transcript of 2.6-kb size was strongly increased, suggesting that the presence of the *aadA* selectable marker does not interfere with the normal processing of *psbD*–*psbC* polycistronic transcripts. In the case of the as-lines, a drastic decrease in *psbD* transcript abundance was observed, indicating that the *aadA* insertion indeed massively reduced transcription from the *psbD* promoter. Only small amounts of the mature dicistronic transcript of 2.6 kb were detectable.

To confirm the identity of the novel large transcripts in the mutants, we used an *aadA*-specific probe (Figure 2, A). As expected, it did not give any signal in the WT, but in the s-mutants revealed bands at 4.8- and 4.0-kb size, which correspond to the mRNA species already detected with the *psbD* probe. A stronger band of 1.8-kb size likely represents the processing product cleaved in front of the *psbD* translation initiation site and still includes the major part of the 5′-UTR of the *psbD* transcript. The strong 1.0-kb signal only covers the *aadA* selectable marker gene terminated by the 3′-*psbA* element downstream of the *aadA* coding region. This band was also the only prominent signal in the as-mutants.

Next, we used a probe specific for the long 5′-UTR of the primary *psbD* transcript (Figure 2, C). As expected, in the WT, this probe allowed us to detect major bands of 4.4- and 3.6-kb size (as reported previously; Yao et al., 1989), likely corresponding to the full-length transcripts covering either only the *psbD*–*psbC* coding regions or additionally including *psbZ*. In the s-mutants, by far the most prominent band was a band of 1.8-kb size, which likely corresponds to the processed *aadA* mRNA transcribed from the *psbD* operon promoter. Finally, we also used a *psbC* probe (Figure 2, D), which resulted in the most complex transcript pattern with more than six distinct bands (Figure 2, E). In the s-mutants, we observed increased accumulation, especially of the full-length transcripts spanning from the selectable marker to *psbZ* and of the processed mRNAs. In the as-mutants, the long transcripts starting from the promoter in front of *psbD* were undetectable. However, shorter transcripts of up to 2.6-kb length starting from the *psbC*-specific promoter within the *psbD* coding region (Yao et al., 1989) accumulated to almost normal levels. These either spanned both *psbC* and *psbZ*, due to incomplete transcription termination at the end of *psbC*, or covered only *psbC*.

### Chloroplast translation in the mutants

**Figure 3:**
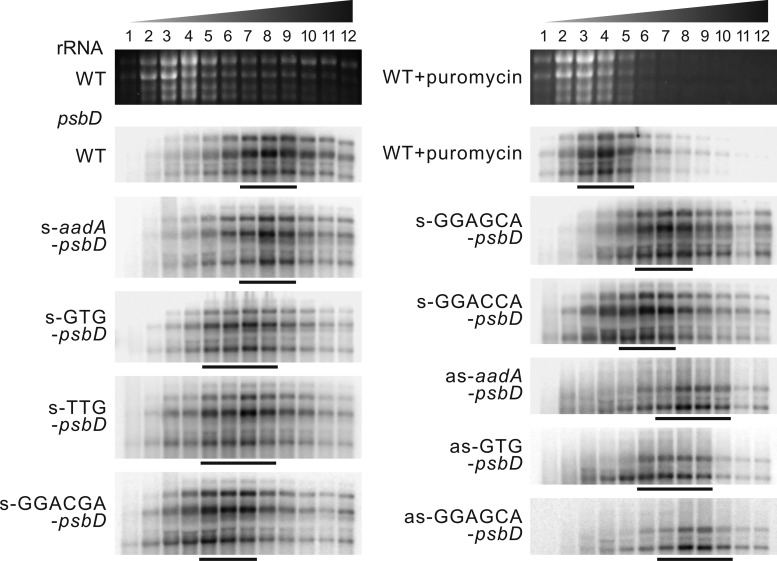
psbD polysome loading analyses of sense and antisense transformants grown on soil. Material was harvested from young leaves with still active thylakoid biogenesis. mRNAs with different ribosome coverage were separated by sucrose density gradient centrifugation into 12 fractions, with fraction 12 being the fraction of highest density. To determine the distribution of free, untranslated mRNAs, a puromycin-treated WT sample was also analyzed. Puromycin dissociates ribosomes from the mRNAs, as confirmed by comparison of ethidium bromide-stained agarose gels (upper panels). The major *psbD*-containing fractions are indicated by horizontal bars below each blot.

To obtain information on changes in *psbD* translation due to the changes in mRNA abundance and the point mutations introduced into the 5′-UTR or the start codon, we performed polysome loading analyses as a proxy for translation activity (Barkan, 1998). Material was harvested from young leaves with still active thylakoid biogenesis. mRNAs with different ribosome loading were separated by sucrose density gradient centrifugation into 12 fractions, with fraction 12 being the fraction of highest density (Figure 3). To determine the distribution of free, untranslated mRNAs, a puromycin-treated WT sample was also analyzed. Puromycin dissociates ribosomes from mRNAs. In the puromycin control, the *psbD–psbC* mRNA was mainly found in fractions 3–5, while in the untreated WT, mRNA distribution peaked in fractions 7–9. Despite the massive increase in mRNA abundance in the s-*aadA–psbD* mutant, mRNA distribution and therefore translation efficiency per mRNA was unaltered, indicating increased total synthesis of PsbD. Both the s-GTG-*psbD* and s-TTG-*psbD* mutants showed a marked shift in mRNA distribution toward fractions of lower density. Point mutations in the SD also shifted mRNA distribution in the polysome profile upward by one–three fractions, and this effect was most pronounced in the s-GGACGA*-psbD* mutant. While the as-*aadA*–*psbD* mutant showed a polysome distribution very similar to the WT, the as-GTG-*psbD* mutant and the as-GGAGCA-*psbD* mutant showed mild shifts in their polysome profiles by one–two fractions toward a lower density.

Resolution and sensitivity of polysome analyses are rather limited, and in the case of polycistronic transcripts, due to the physical linkage of the reading frames, polysome loading only provides an average value of the total translation of the entire transcript (reviewed by Zoschke and Bock, 2018). Therefore, translation of *psbD* and *psbC* cannot be distinguished, and the altered polysome profiles could be due to altered translation of either *psbD* or *psbC*, or both. For example, *psbD* translation might alter translation of *psbC* due to translational coupling (Adachi et al., 2012).

**Figure 4:**
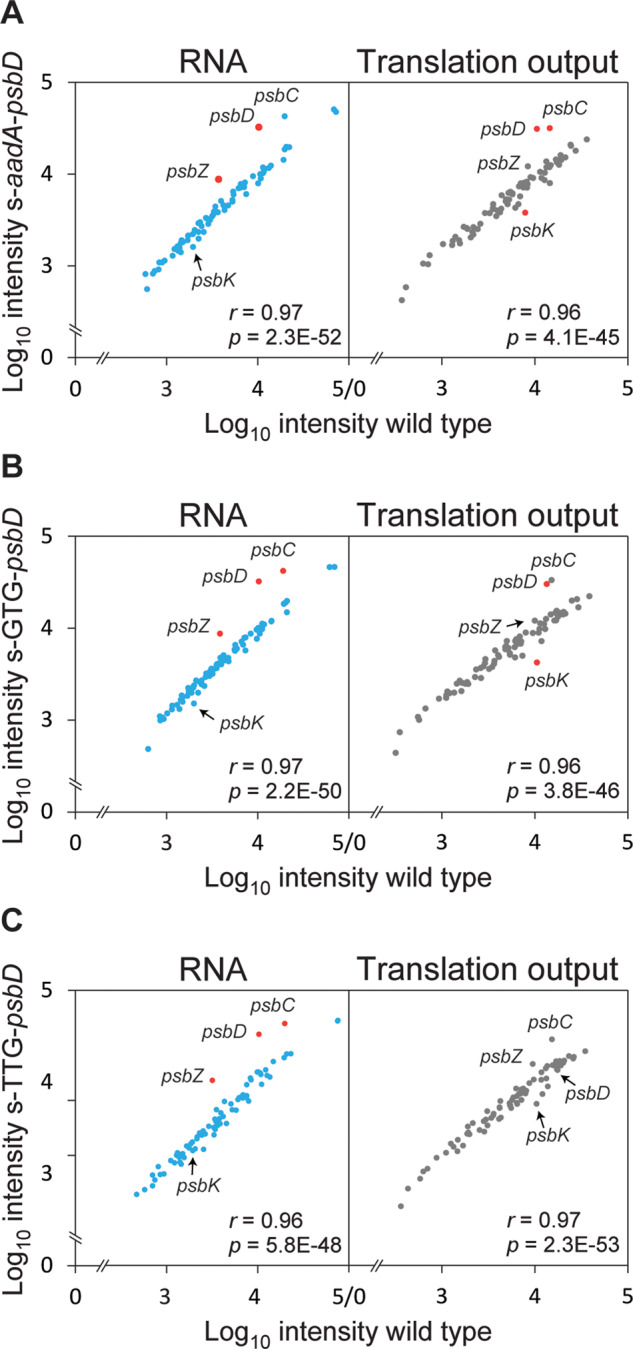
Relative translation output and RNA accumulation levels of transplastomic *psbD* mutant lines. Relative transcript accumulation (RNA) levels and chloroplast ribosome footprint (translation output) were examined by microarray-based ribosome profiling and compared between mutant and WT. Average ribosome footprint and transcript abundances were calculated for each reading frame as described in the “Materials and methods” section, and the mean of three biological replicates was log_10_-transformed and plotted for the transplastomic lines s-*aadA*-*psbD* (**A**), s-*psbD*-GTG (**B**), and s-*psbD*-TTG (**C)** against the corresponding WT (left panels: transcript levels, right panels: ribosome footprints; for better visualization *x*- and *y*-axes are broken). Genes showing more than two-fold changes are highlighted with red data points and named beside the data points in all analyses. Pearson’s *r* and ANOVA’s *P*-value (in nEm non-superscript format for *n* • 10 m) are given within each plot.

To characterize transcription and translation in more detail, we selected the s-*aadA–psbD* control mutant, the two s-translation initiation codon mutants, and the WT for a comprehensive analysis of changes in transcript accumulation and translation of all plastome-encoded genes by transcript and ribosome profiling. To this end, transcript abundances and ribosome footprints were analyzed using high-resolution tiling arrays and then averaged for each chloroplast open reading frame as previously described (Trösch et al. 2018). In each case, ribosomal footprints (“translation output”) and transcript abundances (“RNA”) of the three different mutants were plotted against the signals of WT tobacco analyzed in the same experiment (Figure 4, *x*-axis). To determine the relative translation efficiency of each gene, data were log_2_-transformed and transcript abundance was subtracted from ribosomal footprints (Supplemental Figure S1; Zoschke et al., 2013). The data shown represent averages from three biological replicates (see Supplemental Figure S2). Pronounced changes (more than or equal to two-fold) are highlighted in red color in Figure 4.

When the WT was compared with the s-*aadA*–*psbD* control mutant (Figure 4, A), we observed strong increases in transcript abundance of *psbD* and *psbZ*, in line with the changes observed by northern blot analysis (Figure 2). *psbC* transcript abundance also increased but remained slightly below two-fold. Abundance of all other chloroplast transcripts did not change substantially. The translation output of *psbD* and *psbC*, but not of *psbZ*, clearly increased. Unexpectedly, translation of *psbK* clearly decreased. However, at the level of the relative translation efficiency, not a single pronounced difference between WT and the s-*aadA*–*psbD* control mutant was observed (Supplemental Figure S1). The increased transcript abundance of *psbD* and *psbC* directly resulted in increased translation output, in agreement with their WT-like polysome distribution profiles (Figure 3).

In the s-GTG-*psbD* mutant (Figure 4, B), *psbD*, *psbC*, and *psbZ* mRNA accumulation levels increased more than two-fold. This resulted in a pronounced increase in the translation output of both *psbD* and *psbC*, suggesting that the GUG initiation codon is almost as efficient as the standard AUG translation initiation codon for *psbD*. Similar to the other two s-mutants, the s-TTG-*psbD* mutant displayed clear increases in *psbD*, *psbC*, and *psbZ* transcript abundances (Figure 4, C). However, different from the s-GTG-*psbD* mutant and despite its increased mRNA abundance, the s-TTG-*psbD* mutant did not show a pronounced increase in translation output for any of these reading frames. This is due to a strong reduction in the translation efficiency of especially *psbD*, indicating that the UUG initiation codon is much less efficient than AUG and GUG (Supplemental Figure S1). The strongly diminished translation efficiency of *psbD* did not hamper translation of *psbC*. Consequently, in vivo, *psbC* translation can be considered as uncoupled (or only marginally coupled) to *psbD* translation initiation, contrasting a conclusion that was previously drawn based on *in vitro* translation data (Adachi et al., 2012).

### Only minor differences in photosynthetic parameters of s-mutants

**Figure 5:**
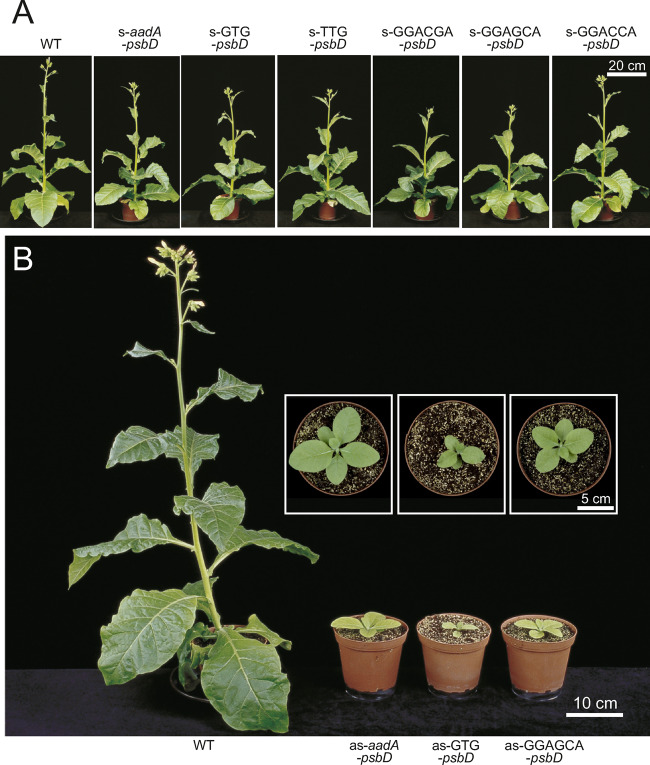
Growth phenotypes of transplastomic *psbD* mutants. (**A**) Growth phenotypes of the s-*psbD* transplastomic lines grown in soil at 350 µE m^−2^ s^−1^. The photographs were taken 10 weeks after seeds had been sown in soil, when the WT plants started to flower (scale bar = 20 cm). (**B**) Growth phenotypes of the as-*psbD* transplastomic lines grown in soil at 100 µE m^−2^ s^−1^. The photographs were taken when the WT plants began to flower (scale bar = 10 cm). The insets show close-up top views of the transplastomic plants (scale bar = 5 cm).

To determine effects of the altered *psbD* transcript accumulation and translation initiation on plant growth and photosynthetic performance, the plants expressing the selectable marker gene in s-orientation were grown in soil under long-day conditions and with an actinic light intensity of 350 µE m^−2^ s^−1^. Plant growth and all physiological parameters analyzed (see below) were not significantly different between independent mutant lines harboring the same construct. Therefore, the growth phenotype of one representative plant per construct (Figure 5, A), and average values of all mutant lines per construct (Table 1) are shown. Growth of all mutants was only slightly retarded relative to that of the WT (Figure 5, A).

**Table 1:** Average values and standard deviation of chlorophyll content, chlorophyll a/b ratio, leaf absorptance, maximum quantum efficiency of PSII in the dark-adapted state (*F*_V_/*F*_M_), and photosynthetic complex contents per leaf area of sense-*psbD* mutants grown at an actinic light intensity of 350 µE m^−2^ s^−1^

Parameter	WT	s-*aadA*–*psbD*	s-GTG-*psbD*	s-TTG-*psbD*	s-GGACGA-*psbD*	s-GGAGCA-*psbD*	s-GGACCA-*psbD*
Sample size	14	13	12	11	12	12	11
Chlorophyll [mg/m^2^]	442.3 ± 98.4	454.7 ± 102.5	436.1 ± 74.6	364.7 ± 66.1	520.9 ± 91.1	519.3 ± 72.1	492.1 ± 42.1
Chlorophyll *a*/*b*	4.04 ± 0.16	**3.84 ± 0.07**	**3.80 ± 0.11**	**3.72 ± 0.13**	**3.85 ± 0.11**	3.85 ± 0.19	**3.82 ± 0.10**
Leaf absorptance (%)	88.5 ± 1.8	88.2 ± 2.2	88.4 ± 2.1	86.9 ± 1.9	89.8 ± 1.2	89.6 ± 1.2	89.3 ± 0.6
*F*_v_/*F*_m_	0.81 ± 0.02	0.80 ± 0.02	0.80 ± 0.02	**0.79 ± 0.01**	0.80 ± 0.02	0.81 ± 0.01	0.80 ± 0.02
PSII [µmol/m^2^]	1.11 ± 0.25	1.16 ± 0.21	1.06 ± 0.17	**0.81 ± 0.20**	1.14 ± 0.17	1.20 ± 0.19	1.05 ± 0.12
Cyt b_6_f [µmol/m^2^]	0.49 ± 0.14	0.45 ± 0.07	0.45 ± 0.12	**0.37 ± 0.10**	0.58 ± 0.12	0.51 ± 0.10	0.52 ± 0.08
Pc [µmol/m^2^]	2.21 ± 0.65	2.55 ± 0.66	2.43 ± 0.59	1.90 ± 0.73	**2.93 ± 0.46**	2.78 ± 0.55	2.83 ± 0.41
PSI [µmol/m^2^]	0.97 ± 0.24	0.99 ± 0.22	0.95 ± 0.16	0.81 ± 0.19	1.15 ± 0.17	1.13 ± 0.18	1.08 ± 0.10

Boldface text highlights values, which are significantly different from WT (*P* > 0.05, one-way ANOVA, Holm–Sidak and Dunn’s method).

**Figure 6:**
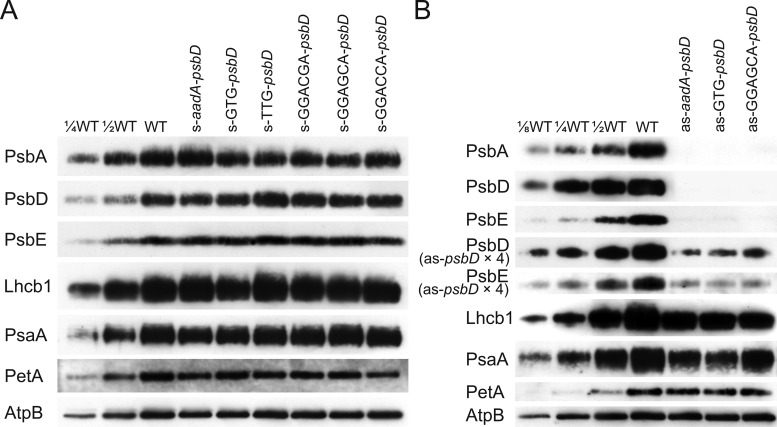
Protein accumulation in the transplastomic lines. (**A**) Immunoblot analysis for s-*psbD* mutants. Immunoblot analyses were performed using antibodies against diagnostic subunits of PSII (PsbA, PsbD, and PsbE subunits), LHC II (LHCB1), photosystem I (PsaA), the cytochrome b_6_f complex (PetA, i.e. cytochrome f), and the chloroplast ATP synthase (AtpB). Samples were loaded on an equal leaf area basis. For the WT, a dilution series is included to enable semiquantitative assessments. (**B**) Immunoblot analysis for as-*psbD* mutants. As accumulation of the PSII subunits was barely detectable in the as-*psbD* transplastomic lines, four times of the thylakoid amounts in these as-*psbD* lines (as-*psbD* × 4) were loaded along with unchanged amounts of WT samples.

To determine possible changes in the composition and function of the photosynthetic apparatus in detail, the chlorophyll a/b ratio, the chlorophyll content per leaf area, leaf absorptance, and the maximum quantum efficiency of PSII in the dark-adapted state (*F*_V_/*F*_M_) were determined for the youngest fully expanded leaves of plants at the onset of flowering (Table 1). Then, thylakoids were isolated from these leaves and the contents of both photosystems, the cyt b_6_f and plastocyanin (Pc), were determined spectroscopically from chemically or light-induced difference absorbance signals of cyt b_559_ (PSII), cytochromes b_6_ and f (cyt b_6_f), and P_700_ (PSI; see the “Materials and methods” section). Finally, these data were re-normalized to a leaf area basis (Table 1). These quantifications were validated by immunoblots against essential subunits of the different photosynthetic complexes (Figure 6).

Neither chlorophyll content nor leaf absorptance differed significantly between WT and the s-mutants, while the chlorophyll a/b ratio of all mutants was slightly lower than in the WT (Table 1). The maximum quantum efficiency of PSII in the dark-adapted state (*F*_V_/*F*_M_), as well as the PSII and cyt b_6_f content per leaf area were only slightly lower in the s-TTG-*psbD* mutant than in the WT. In these parameters, all other mutants did not differ from the WT. For PSI accumulation, no significant differences could be observed for any of the s-mutants. These results were confirmed by immunoblots against the essential PSII RC core subunits PsbA (D1), PsbD (D2), and PsbE, a subunit of cyt b_559_. LHCB1 accumulation did not reveal any differences between the WT and any of the s-mutants. When PSI, the cyt b_6_f, and chloroplast ATP synthase were probed with antibodies against their essential subunits PsaA, PetA (cytochrome f), and AtpB, no obvious differences in protein accumulation between mutants and WT could be observed (Figure 6), in line with the spectroscopic results.

**Figure 7:**
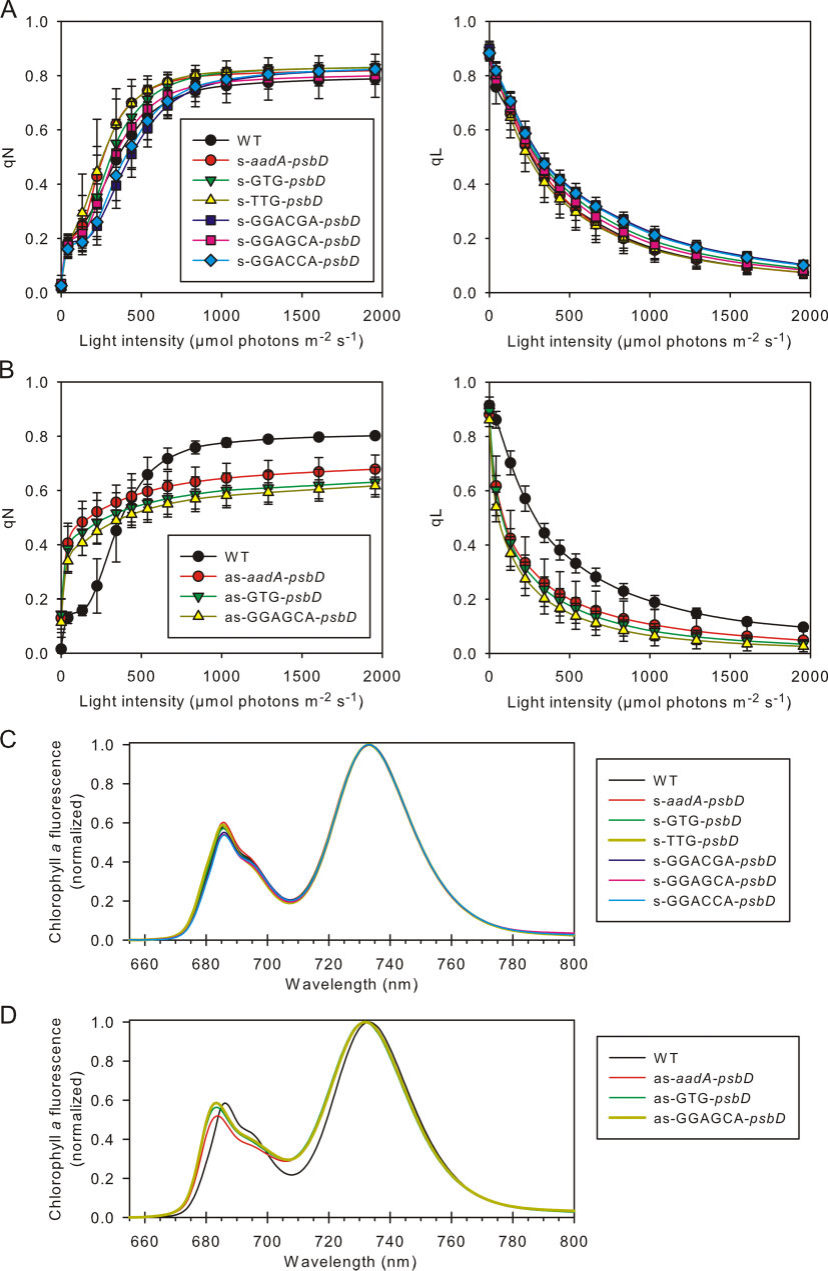
Chlorophyll-a fluorescence analysis of transplastomic *psbD* mutants. (**A**) and (**B**) Light response curves of qL (redox state of the PSII acceptor sides) and qN (non-photochemical quenching) in the s-*psbD* (A) and as-*psbD* (B) transplastomic lines. Error bars indicate the standard deviation of the mean, and the sample size is the same as indicated in Table 1. (**C**) and (**D**) 77K chlorophyll-a fluorescence emission spectra in the s-*psbD* (C) and as-*psbD* (D) transplastomic lines. The spectra are averaged per construct (n indicated in Table 1) and normalized to the PSI emission maximum at ∼735 nm.

Accordingly, most mutants displayed only subtle changes in the light response curves of the chlorophyll-a fluorescence parameters qL (a measure for the redox state of the PSII acceptor side; Kramer et al., 2004) and qN (a measure for the thermal dissipation of excess excitation energy in the PSII antenna system, Krause and Weis, 1991). Again, average values of all mutant lines per construct are shown (Figure 7). In the s-TTG-*psbD* mutant, the only s-mutant displaying a significant decrease in PSII accumulation, induction of qN was slightly shifted to lower light intensities, while for the reduction state of the PSII acceptor side, no significant differences were observed (Figure 7, A). Finally, 77K chlorophyll-a fluorescence emission spectra were recorded and normalized to the PSI emission signal at 734-nm wavelength (Figure 7, C). Again, no changes in the maximum emission signal of PSII at 686.5-nm wavelength were observable, strongly suggesting that the LHCII antenna proteins were well coupled to the RC, even in the s-TTG-*psbD* mutant.

### As-mutants suffer from massive growth retardation and impaired photosynthesis

All three autotrophically viable as-mutants showed delayed growth and evidence of reduced photosynthetic performance. They were highly light-sensitive when grown at 350 µE m^−2^ s^−1^, the light intensity used for the characterization of the s-mutants. Therefore, the as-mutants were grown at a reduced light intensity of 100 µE m^−2^ s^−1^. Photographs in Figure 5, B were taken when the WT started to flower. At that time, all three mutants were strongly retarded in growth, most severely the as-GTG-*psbD* mutant. For a more detailed physiological analysis, and to avoid artifacts due to differences in the developmental state of the plants, we used young fully expanded source leaves of non-flowering WT plants and mutants (Table 2). Despite the reduced growth light intensity, the data for WT tobacco are almost indistinguishable from those of the WT grown at 350 µE m^−2^ s^−1^. This is unsurprising because, between 100 and 350 µE m^−2^ s^−1^, the photosynthetic apparatus of tobacco only shows minor responses to the growth light intensity (Schöttler et al., 2017).

**Table 2:** Average values and standard deviation of chlorophyll content, chlorophyll a/b ratio, leaf absorptance, maximum quantum efficiency of PSII in the dark-adapted state (*F*_V_/*F*_M_), and photosynthetic complex contents per leaf area of as-*psbD* mutants grown at a reduced actinic light intensity of 100 µE m^−2^ s^−1^

Parameter	WT	as-*aadA*–*psbD*	as-GTG-*psbD*	as-GGAGCA-*psbD*
Sample size	8	16	10	8
Chlorophyll [mg/m^2^]	413.9 ± 45.6	**145.2 ± 19.7**	**128.5 ± 11.7**	**124.7 ± 26.6**
Chlorophyll *a*/*b*	4.18 ± 0.11	**3.79 ± 0.15**	**3.94 ± 0.09**	**3.83 ± 0.09**
Leaf absorptance (%)	87.7 ± 1.1	**73.0 ± 2.8**	**71.3 ± 2.5**	**70.4 ± 3.3**
*F*_v_/*F*_m_	0.80 ± 0.01	**0.28 ± 0.07**	**0.25 ± 0.02**	**0.24 ± 0.05**
PSII [µmol/m^2^]	1.19 ± 0.14	**0.22 ± 0.05**	**0.23 ± 0.05**	**0.19 ± 0.02**
Cyt b_6_f [µmol/m^2^]	0.51 ± 0.11	**0.29 ± 0.05**	**0.24 ± 0.10**	**0.31 ± 0.08**
Pc [µmol/m^2^]	2.27 ± 0.41	**1.24 ± 0.32**	**1.35 ± 0.25**	**1.19 ± 0.19**
PSI [µmol/m^2^]	1.01 ± 0.11	**0.34 ± 0.05**	**0.32 ± 0.03**	**0.31 ± 0.07**

Boldface text highlights values, which are significantly different from WT (*P* > 0.05, one-way ANOVA, Holm–Sidak and Dunn’s method).

All parameters tested were significantly different between the WT and the three as-mutants. The chlorophyll content per leaf area in the as-mutants was reduced to less than 35% of the WT level. Accordingly, leaf absorptance decreased as well. The reduction in the chlorophyll a/b ratio was less drastic, indicating a global reduction of all components of the photosynthetic apparatus, instead of a selective loss of PSII. Specific impairments in PSII RC accumulation should result in a strong decrease of the chlorophyll a/b ratio, because RCs only bind chlorophyll a, while the LHCs of the photosystems bind both chlorophyll a and b. The maximum quantum efficiency of chlorophyll-a fluorescence was drastically reduced in all three as-mutants, suggesting PSII photoinhibition or the presence of uncoupled LHC in the thylakoid membrane. In line with the strongly reduced chlorophyll content and the only moderately affected chlorophyll a/b ratio, the accumulation of all photosynthetic complexes per leaf area was strongly reduced: PSII contents decreased to less than 20% of WT levels in all three mutants, and also PSI accumulation was severely reduced to approximately 30% of WT levels. The pronounced effect of reduced PSII contents on PSI accumulation is unsurprising, in that similar findings have been reported for Arabidopsis antisense mutants against the PSBO subunit of the OEC (Dwyer et al., 2012). The reduction in both photosystems was more pronounced than that in the total chlorophyll content, indicating that LHC accumulation was affected to a lesser extent. Also, the cyt b_6_f and Pc were less strongly affected than the photosystems, decreasing only by 40%–50% in content, relative to the WT. Changes in photosynthetic complex accumulation were again confirmed immunologically (Figure 6, B). Again, as proxies for PSII accumulation, PsbA (D1), PsbD (D2), and PsbE were probed. Accumulation of all PSII subunits strongly decreased. In line with the other redox-active complexes being less affected than PSII, accumulation of PsaA (PSI) and PetA (cyt b_6_f) was less severely reduced than that of the PSII subunits.

On a functional level, the light response curves of qL and qN revealed pronounced differences between the WT and the as-mutants. Both the induction of qN and the reduction of the PSII acceptor side were strongly shifted to lower light intensities, while the full induction of qN in saturating light was impaired in the as-mutants (Figure 7, B). Finally, in the 77K chlorophyll-a fluorescence emission spectra, the maximum emission wavelengths of PSI-LHCI and especially of PSII-LHCII were clearly shifted to lower wavelengths, indicating the presence of free, uncoupled LHC (Figure 7, D). Uncoupled LHCI emit fluorescence at 77K with maxima between 701 and 730 nm (Castelletti et al. 2003; Croce et al., 2004), while the coupled LHCI-PSI in the WT show their typical emission maximum at 734 nm. Likewise, the shift of the PSII-LHCII emission maximum from 686.5 to 682 nm indicates the presence of free, uncoupled LHCII, whose emission maximum is at 680 nm (Krause and Weis, 1991).

## Discussion

The biogenesis of PSII has been studied for decades, and its assembly sequence is well established. Also, more than 20 auxiliary proteins stabilizing different assembly intermediates and supporting the insertion of pigments and co-factors have been identified (Nickelsen and Rengstl, 2013; Lu, 2016). By contrast, much less is known about the limiting steps of PSII biogenesis, and how PSII accumulation is adjusted to different growth light intensities, which can result in more than four-fold changes in PSII content (reviewed by Schöttler and Tóth, 2014). Therefore, identifying the rate-limiting step(s) of PSII biogenesis is of major importance to better understand the acclimation of the photosynthetic apparatus to different environmental conditions, and might pave the way for targeted manipulations of PSII content, without altering its subunit composition and function.

Here, we have addressed a potential limiting role of the two plastome-encoded subunits D2 and CP43, which are expressed from a single operon-type transcription unit in tobacco. Our main focus was on D2, which together with cyt b_559_ initiates the PSII assembly process. We did not consider cyt b_559_ as a candidate for controlling PSII accumulation, because much higher amounts of cyt b_559_ than of functional PSII accumulate in Arabidopsis mutants with massively disturbed primary metabolism (Schmitz et al., 2012). In *C. reinhardtii*, a reduction in *psbD* mRNA directly results in a proportional reduction in D2 protein and PSII abundance, pointing to an important regulatory role of *psbD* transcript accumulation (Klinkert et al., 2005). Mutations in a repressor element of translation initiation increase not only *psbD* translation initiation efficiency, but also PSII accumulation by up to 20% (Klinkert et al., 2006). A similar overaccumulation of functional PSII by increased synthesis of D2 (either due to increased *psbD* mRNA abundance or increased rates of translation) would be the ultimate proof that, similar to the situation in *C. reinhardtii*, D2 is also limiting PSII biogenesis in vascular plants.

### Neither D2 nor CP43 limits PSII accumulation in tobacco

To generate transplastomic tobacco with altered *psbD* transcript abundance, we disrupted the *psbD* 5′-UTR via insertion of the selectable marker gene *aadA*. In the s-mutants, read-through transcription from the strong *Prrn* promoter of the *aadA* massively increased *psbD* (and *psbC*) transcript abundance (Figure 2). This increase in *psbD* mRNA levels was further confirmed by the chloroplast transcriptome profiling data (Figure 4), which additionally showed a strong increase in *psbZ* mRNA abundance. This again is a consequence of transcriptional read-throughs through the *psbD*–*psbC* dicistron into *psbZ* located further downstream. In the as-mutants, whose strongly decreased *psbD* transcript abundance is similar to the reduced *psbD* transcript accumulation in the *C. reinhardtii* promoter mutant (Klinkert et al., 2005), the massive decrease in transcript abundance resulted in a more than 80% reduction in PSII accumulation (Table 2). This could not be compensated by increased translation of the residual mRNAs (Figure 3). This observation is in line with a limiting role of D2 synthesis for PSII assembly.

However, the strong increase in *psbD* and *psbC* transcripts in the s-*aadA–psbD* mutant did not result in increased PSII accumulation, even though mRNA distribution in the sucrose density gradients used for polysome analysis remained unchanged, indicating that the supernumerary mRNA molecules undergo translation (Figure 3). This conclusion is also supported by ribosome footprint analyses, which show proportional increases of both transcript abundance and translation output for *psbD* and *psbC* (Figure 4, A). Consequently, relative translation efficiency remained unaltered (Supplemental Figure S1). However, neither D2 protein level (Figure 6, A) nor PSII content (Table 1) increased. Also, all other photosynthetic parameters remained unaltered (Table 1). Light response curves of qN and qL did not reveal any major differences to the WT (Figure 7, A). Likely, due to limitation of PSII biogenesis by another so far unknown factor, the additionally synthesized D2 and CP43 are condemned to rapid degradation. Despite the increased transcript abundance and translation of *psbD*, *psbC*, and *psbZ*, the transcript abundance and translation of both *psbA* and *psbB* remained unaltered. Also, for the other plastome-encoded subunits of PSII, no obvious changes in transcript abundance or translation were observed in response to the increased transcription and translation of *psbD* and *psbC*, except for a slightly reduced translation of *psbK* (see below). Clearly, no feed-forward stimulation of PSII biogenesis by the synthesis of supernumerary D2 and CP43 subunits exists in tobacco.

In *C. reinhardtii*, it is still unclear how the increased *psbD* translation initiation resulted in increased PSII accumulation (Klinkert et al., 2006). A negative regulatory mechanism called “Control by Epistasy of Synthesis” (CES), by which the absence of D2 represses the synthesis of other plastome-encoded subunits of PSII, has been characterized in detail (Minai et al., 2006): D2 (or, more likely, the D2-cyt b_559_ subcomplex) is required for efficient translation of D1, because unassembled D1 inhibits its own translation. Different to the situation in *Synechocystis* PCC6803, where the inner antenna proteins accumulate independent of the RC subunits (Pakrasi et al., 1989; Komenda et al., 2004; Boehm et al., 2011), CP47 is also part of this regulatory circuit. The presence of the “RC-like complex” as acceptor for newly synthesized CP47 is a prerequisite for the translation of *psbB*, because again, unassembled CP47 blocks its own synthesis (Minai et al., 2006). Thereby, CES is a highly efficient negative feedback mechanism avoiding the wasteful accumulation of supernumerary RC subunits in the absence of either D2 or D1 as assembly partners. Potentially, via this mechanism, increased abundance of D2 and the D2-cyt b_559_ subcomplex might stimulate the synthesis of D1, and the formation of the RC-like complex, in turn, might stimulate translation of CP47 by allowing its assembly into the nascent PSII complex. However, so far, in *Chlamydomonas*, such a feed-forward activation of translation has not been demonstrated. While our data reported here clearly establish that a feed-forward activation of PSII biogenesis by a CES-like process does not exist in tobacco, this does neither rule out the existence of such a regulatory loop in *C. reinhardtii*, nor does it exclude the possibility of a feedback inhibitory CES-like process in vascular plants. More detailed studies on plants with diminished synthesis of the D2 protein, including the as-*psbD*-mutants introduced here, are needed to clarify these questions.

Another important conclusion from the analysis of the s-*aadA*–*psbD* mutant is that, in tobacco, translation of *psbD* and *psbC* is either independent of mRNA-specific translation initiation factors, or these are present in large excess. If any such factor were limiting, overexpression of the transcript would have resulted in lower translation efficiency. Chloroplast mRNAs with an SD in their 5′-UTR may be less dependent on mRNA-specific translation factors than those that do not contain a SD (Scharff et al., 2017; Zoschke and Bock, 2018). Alternatively, because translation of *psbK* was slightly decreased in the s-*aadA–psbD* and the s-GTG-*psbD* mutant (Figure 4), a specific translation factor might be shared between *psbD* (and/or *psbC*) and *psbK*, possibly explaining its decreased translation.

### Translation initiation mutants

In addition to increasing and decreasing the mRNA abundance of the operon genes, we altered *psbD* translation initiation efficiency via the introduction of point mutations into the initiation codon. This approach had been previously applied to reduce accumulation of chloroplast ATP synthase by the introduction of point mutations into the start codon of the catalytic AtpB subunit (Rott et al., 2011), and to repress the essential plastid-encoded ClpP subunit of the stromal Clp protease (Moreno et al., 2017). While the GTG-*atpB* mutation had a more severe effect on ATP synthase accumulation than the TTG-*atpB* mutation (Rott et al., 2011), in the case of ClpP, the TTG-*clpP* mutation resulted in a more severe defect (Moreno et al., 2017). Here, ribosome footprint analysis (Figure 4) revealed that the GUG initiation codon was almost as efficient as the AUG start codon for *psbD* (Supplemental Figure S1). Translation efficiency of *psbD* in the s-TTG-*psbD* mutant was strongly reduced, showing that the UUG initiation codon is less efficient than either AUG or GUG in the sequence context of the *psbD* 5′-UTR (Supplemental Figure S1). Accordingly, different from all other s-mutants, the s-TTG-*psbD* mutant suffered from a mild reduction of PSII content (Table 1). Furthermore, the as-GTG-*psbD* mutant was viable under autotrophic conditions, while the as-TTG-*psbD* mutant was not, again suggesting that translation initiation from the TTG codon was less efficient. Finally, despite the strongly diminished translation efficiency of *psbD* in the s-TTG-*psbD* mutant, translation of *psbC* remained unaffected, arguing against a strong coupling of *psbC* translation initiation and termination of *psbD* translation, which had been previously suggested based on *in vitro* studies (Adachi et al., 2012).

We also generated mutants with an altered SD, because *psbD* translation was severely affected in transplastomic tobacco plants harboring a mutation in the anti-SD (Scharff et al., 2017). Overall, the behavior of the SD mutants was very similar to the start codon mutants. The s-SD-*psbD* mutants were similar to the s-GTG-*psbD* mutant, in that no differences to the WT were observable (Table 1 and Figures 5–7).The as-GGAGCA-*psbD* mutant was most similar to as-GTG-*psbD*, while the other two as-SD-*psbD* mutants did not survive under autotrophic conditions, similar to the as-TTG-*psbD* mutant.

### What limits PSII biogenesis in vascular plants?

In cyanobacteria and green alga, most data point to a critical role of D2 synthesis in controlling the biogenesis of PSII. In the Δ*psbEFLJ* mutant of the cyanobacterium *Synechocystis* PCC6803, translation of *psbD* is abolished, suggesting a direct role of cyt b_559_ in regulating D2 synthesis (Komenda et al., 2004). However, in addition to the fraction of cyt b_559_ bound to PSII, cyt b_559_ is also part of a complex containing no other subunits of PSII. Therefore, cyt b_559_ is unlikely to be the only limiting factor for *psbD* translation and PSII accumulation. D1 is translated in the absence of both cyt b_559_ and D2 but is rapidly degraded again unless it is stabilized by association with the D2-cyt b_559_ subcomplex (Komenda et al., 2004), suggesting that D2 is the major limiting factor for PSII assembly and that cyt b_559_ is one factor controlling the synthesis of D2, while the downstream assembly partner D1 does not exert a similar control over *psbD* translation (Komenda et al., 2004). Different to the situation in *Synechocystis* PCC6803, in *C. reinhardtii*, cyt b_559_ does not control translation of *psbD* (Morais et al., 1998). Because a clear increase in *psbD* translation due to the mutation of a repressor element of translation initiation results in increased accumulation of PSII, D2 synthesis seems to control PSII accumulation (Klinkert et al., 2006).

Because of the previous observation that free cyt b_559_ can accumulate in vascular plants, and therefore is likely not the limiting factor for PSII biogenesis (Schmitz et al., 2012), and because our data eliminate both D2 and CP43 as limiting factors for PSII accumulation, the rate-limiting step in PSII biogenesis seems to have changed several times during the evolution of oxygenic photosynthesis. For vascular plants, it still remains to be identified. A limiting function of one of the other plastome-encoded subunits of PSII appears unlikely, given that a rate-limiting step should occur early in the pathway. The next subunits to assemble into the D2-cyt b_559_ subcomplex are D1 and the small PsbI subunit. PsbI is not essential for PSII accumulation (Schwenkert et al., 2006). In vascular plants, for multiple reasons, D1 is an unlikely candidate to control PSII biogenesis. First, an autoregulatory loop may adjust the synthesis of D1 to the presence of assembly partners (Chotewutmontri and Barkan, 2020): Accumulation of D1 in an assembly complex blocked *psbA* translation, possibly due to repressive interactions with translational activators of *psbA*. In the absence of the auxiliary protein High Chlorophyll Fluorescence136 (HCF136), which may associate newly translated D1 with this assembly complex, ribosome occupancy of *psbA* is always high. This autoregulation of D1 synthesis strongly suggests that another assembly step prior to the insertion of D1 limits PSII accumulation, and that D1 synthesis is adjusted to the capacity of this reaction.

Furthermore, because PSII is prone to photodamage and damaged D1 needs to be rapidly replaced in the PSII repair cycle, this could generate the problem to independently regulate D1 synthesis for the PSII repair cycle and for de novo assembly of PSII (reviewed by Mamedov and Styring, 2003; Järvi et al., 2015). The situation is different in cyanobacteria, where de novo biogenesis and repair of PSII may be spatially separated: The earliest steps of PSII assembly in *Synechocystis* PCC6803 occur at “thylakoid centers,” where thylakoids and the plasma membrane are in close contact (Stengel et al., 2012; Nickelsen and Rengstl, 2013), while PSII repair may occur in distinct repair zones in the thylakoid membrane (Sacharz et al., 2015). In the chloroplast of *C. reinhardtii*, de novo biogenesis of both PSII and PSI occurs in specialized “translation zones,” while PSII repair is distributed over the unstacked stroma lamellae (Uniacke and Zerges, 2007; Sun et al., 2019). In vascular plants, which display strong lateral heterogeneity of stacked grana thylakoids and unstacked stroma lamellae, no evidence exists for distinct thylakoid domains similar to “thylakoid centers” or “translation zones.” Instead, the first steps of both de novo biogenesis and PSII repair take place in the stroma lamellae, with PSII then moving toward the grana core during the later steps of assembly and photoactivation (Mamedov et al., 2000; Mamedov et al., 2008).

Among all subunits incorporated at a later point into nascent PSII, our work rules out a limiting function of CP43, leaving CP47 as the only so far uninvestigated candidate among the major subunits. Besides CP47 and the luminal subunits attached late in the PSII assembly process (prior to dimerization and supercomplex formation; Shevela et al., 2016), only low molecular mass subunits would remain as potential candidates for a limiting role. Among these, only PsbH is essential for PSII accumulation in *C. reinhardtii* (Summer et al., 1997). All other small subunits are not absolutely essential for PSII accumulation, and knock-out mutants only suffer from reduced PSII accumulation and/or impaired activity, such as transplastomic Δ*psbJ* and Δ*psbL* mutants in tobacco (Regel et al., 2001; Hager et al., 2002; Ohad et al., 2004), and the Δ*psbK* mutant in *C. reinhardtii* (Takahashi et al., 1994). Other mutants affected in small subunits of PSII suffer from milder functional defects (reviewed by Shi and Schröder, 2004: Shi et al., 2012; Plöchinger et al., 2016). Furthermore, because the translational autoregulation of *psbA* synthesis suggested that an earlier step in PSII biogenesis is highly regulated, and D1 synthesis is adjusted to it, attempts to stepwise up- and down-regulate the synthesis of the small subunits do not seem to deserve a high priority in identifying the rate-limiting step of PSII biogenesis.

In summary, a rate-limiting function of the subunits of cyt b_559_, or of D2 and CP43, can be excluded for vascular plants, and all available data also argue against a rate limitation at the level of D1 synthesis. Therefore, it might be appropriate to consider a limiting function of either pigment biosynthesis and/or auxiliary proteins. For example, the plastid-encoded assembly chaperone Ycf3 can restrict PSI biogenesis (Petersen et al., 2011; Schöttler et al., 2011). However, several auxiliary proteins are also shared by de novo assembly and PSII repair (Järvi et al., 2015), potentially causing a problem similar to that of D1 synthesis in regulating PSII repair versus de novo assembly. An exception may be auxiliary proteins supporting very early steps of assembly, especially in connection with membrane insertion of apoproteins and chlorophyll binding to the nascent polypeptides. In *Synechocystis* PCC6803, binding of pigments and cofactors during membrane insertion of the nascent polypeptides of photosystem subunits is supported by the formation of a supercomplex consisting of chlorophyll synthase, the PSII assembly factor Ycf39, and the thylakoid insertase Albino3 (Chidgey et al., 2014). Furthermore, in the photosynthesis-deficient mutant 68 (Pam68), a complex of ribosomes, the nascent CP47 protein, and the SecY translocase (required for membrane insertion of CP47) is formed and likely facilitates chlorophyll insertion into the apoprotein (Bučinská et al., 2018). In vascular plants, the one-helix proteins OHP1 and OHP2 together with the scaffold protein High Chlorophyll Fluorescence244 (HCF244) deliver pigments to D1 (Hey and Grimm, 2020). In the absence of either OHP1 or OHP2, synthesis of both D1 and D2 and formation of the PSII RC are blocked (Li et al., 2019). On the other hand, in a chlorophyll-deficient maize (*Zea mays*) mutant, the translation of plastid mRNAs for chlorophyll-binding apoproteins of both photosystems remained unaffected. However, without stabilization by chlorophyll binding, apoproteins undergo rapid co- or post-translational proteolysis (Zoschke et al., 2017). Together, these data may point to a crucial role of the OHPs in coupling pigment biosynthesis and translation of *psbA* and *psbD*.

Therefore, in addition to further exploring possible limiting roles of structural subunits in PSII biosynthesis, the function of chlorophyll channeling into the PSII assembly machinery and its coupling with translation especially of D2 also need to be seriously considered as potential rate-limiting processes. The ultimate proof that a subunit or an auxiliary protein indeed limits PSII biogenesis can only be provided by establishing a strict correlation between its content and PSII accumulation over a wide concentration range, including the demonstration that overexpression of the factor leads to overaccumulation of functional PSII, as reported for *C. reinhardtii* (Klinkert et al., 2006). Also, such a correlation should be validated under different growth conditions. For example, overexpression of the *petA* mRNA increases cyt b_6_f accumulation only in low-light conditions. In high light or under adverse growth conditions such as heat or chilling stress, no difference in cyt b_6_f content between the WT and the *petA* mRNA overexpressor is discernable (Schöttler et al., 2017).

The identification of the bottleneck in PSII biogenesis may also pave the way to targeted manipulations of PSII content, without interfering with PSII subunit composition and function. The latter has been observed in many mutants with decreased PSII contents due to the repression of individual subunits. Most of these mutants contain less PSII, but the residual PSII is functionally impaired (reviewed by Shi et al., 2012; Plöchinger et al., 2016). By specifically manipulating the rate-limiting step of the assembly process, only fully functional PSII of WT structure should accumulate. Such mutants would be excellent tools for basic research on photosynthesis, but also would open up new opportunities in biotechnology and plant breeding.

## Material and methods

### Plant material and growth conditions

Tobacco (*N. tabacum* cv Petit Havana) plants were grown under aseptic conditions on agar-solidified Murashige and Skoog medium containing 30 g/L sucrose (Murashige and Skoog, 1962). Transplastomic lines were rooted and propagated on the same medium. For seed production, transplastomic plants were grown in soil under standard greenhouse conditions. Inheritance and seedling phenotypes were analyzed by germination of surface-sterilized seeds on Murashige and Skoog medium containing 500 mg/L spectinomycin. For photosynthesis measurements and molecular analyses, seeds of tobacco WT and transplastomic plants were germinated under long-day conditions (16-h light) at a light intensity of 100 μE m^−2^ s^−1^. The day temperature was 22°C, the relative humidity 75%. During the night, temperature and relative humidity were decreased to 18°C and 70%, respectively. Four weeks after germination or later (in the case of mutants severely retarded in growth, see the “Results” section), plants were transferred to a Conviron chamber with 350 μE m^−2^ s^−1^ light intensity. All other environmental parameters were kept constant. After a minimum time of 14 d to allow full acclimation to the new environment, all measurements were performed on the youngest fully expanded leaves at the onset of flowering.

### Vector construction for plastid transformation

The region of the tobacco plastid genome containing a part of the *psbD* gene was isolated as a 1.5-kbp KpnI/SacI fragment (Figure 1, A) with primers PsbD_F and PsbD_R (Supplemental Table S1), and then cloned into the pBC SK+ plasmid vector (Stratagene). The resulting construct was used to produce the translation initiation codon mutations by PCR. The region surrounding the SD and the ATG start codon was cleaved with BsaHI and EcoO109I (Figure 1, A) and replaced by PCR products carrying the point mutations. The mutations of the SD or the start codon were introduced with one of the five primers with mutated sites (PsbD_mut1_F to PsbD_mut5_F) and primer PsbD_mut_R as the reverse primer for all the PCR amplifications (Supplemental Table S1). A chimeric *aadA* gene fused to chloroplast-specific expression signals and conferring resistance to the aminoglycoside antibiotics spectinomycin and streptomycin (Svab and Maliga, 1993) was cloned into a unique NdeI site within the 5′-UTR of the *psbD* gene (Figure 1, A) to enable selection of transplastomic lines. Digestion of the final transformation vectors with restriction enzyme KpnI was used to identify the orientations of *aadA* insertion. Both the sense and the antisense orientation of the *aadA* relative to *psbD* were used for plastid transformation.

### Generation of transplastomic plants

Young sterile leaves of tobacco cultivar Petit Havana were bombarded with the respective transformation vectors bound to gold particles (0.6-µm diameter) using a helium-driven biolistic gun (PDS-1000 He; Bio-Rad). Primary transformants were selected from 5 × 5-mm leaf pieces exposed to plant regeneration medium containing 500 mg/L spectinomycin. Several independent transplastomic lines were then subjected to a maximum of three additional rounds of regeneration on spectinomycin-containing medium to enrich the transplastome and select against residual copies of the WT plastome. Spontaneous spectinomycin-resistant plants were eliminated by a double resistance test on medium supplemented with 500 mg/L spectinomycin and 500 mg/L streptomycin (Svab and Maliga, 1993; Bock, 2001; Bock, 2015). Homoplasmic transplastomic lines were transferred to the greenhouse for seed production. The homoplasmic state of the progeny was confirmed by inheritance test and RFLP analysis (Figure 1, B and C**)**. The presence of the mutations was confirmed by DNA sequencing. It should be noted that the as-*aadA*–*psbD* mutant had been previously used as a control by Krech et al. (2013).

### Isolation of nucleic acids and hybridization procedures

Total plant DNA was isolated from fresh leaf material by a rapid cetyltrimethylammoniumbromide-based miniprep procedure (Doyle and Doyle, 1990). DNA samples digested with restriction enzymes were separated in 1% w/v agarose gels and blotted onto Hybond-XL nylon membranes (GE Healthcare) according to the manufacturer’s instructions. RNA was extracted using the peqGold TriFast reagent (Peqlab). RNA samples were separated in 1% formaldehyde-containing agarose gels and blotted onto Hybond-XL nylon membranes. Hybridization probes were generated by PCR amplification using specific oligonucleotides (Supplemental Table S1). Prior to labeling, DNA fragments were purified by agarose gel electrophoresis followed by extraction from an excised gel slice using a NucleoSpin Extract II kit (Macherey-Nagel). Hybridization probes were labeled with α[^32^P]dCTP by random priming (Multiprime DNA labeling kit; GE Healthcare). Hybridizations were performed at 65°C in Rapid-Hyb buffer (GE Healthcare) according to the manufacturer’s instructions. Hybridization signals were quantified using a Typhoon Trio+ variable mode image (Amersham Biosciences) and Image Quant 5.2 software.

### Polysome loading assays

Isolation of polysomes and RNA extraction from sucrose gradient fractions were performed as described previously (Barkan, 1998; Rott et al., 2011). Equal aliquots of extracted RNAs from each fraction were separated by denaturing agarose gel electrophoresis as described above. For the puromycin control, polysome samples with 0.5 mg/mL puromycin were incubated at 37°C for 10 min prior to ultracentrifugation.

### Ribosome profiling

Ribosome footprints and total RNA were isolated as described in Zoschke et al. (2013), except that after micrococcal nuclease treatment to degrade nuclease-accessible mRNA and cleave polysomes into monosomes, 4-mL lysate was layered onto a 1-mL sucrose cushion (30% [w/v] sucrose, 0.1-M KCl, 40-mM Tris–acetate, pH 8.0, 15-mM MgCl_2_, 5-mM 2-mercaptoethanol, 100 µg/mL chloramphenicol, and 100 µg/mL cycloheximide) and centrifuged for 1.5 h at 50,000 rpm at 4°C in a SW55 Ti rotor (Beckman). RNA labeling was performed according to Zoschke et al. (2013) with the following minor modification: 4-µg purified footprints and 3.5-µg fragmented total RNA derived from mutant and WT plants were differentially labeled with Cy3 and Cy5 (ULS Small RNA Labeling Kit, Kreatech Diagnostics), respectively, following the manufacturer’s instructions.

Ribosome and transcriptome profiling data were analyzed as described in Trösch et al. (2018). Briefly, all local background-subtracted single-channel signals (F635-B635 and F532-B532, respectively) were normalized to the average signal of the datasets of the three mutants and their corresponding WT including all replicates of ribosome footprints and total mRNA to remove biases introduced by technical variation. The average of the probe signals for each reading frame (RF) was then log_2_-transformed. The relative abundance of ribosome footprints and total mRNA were calculated for each RF by normalization of the average of each RF to the average signal of all RFs. By doing so we obtained for each RF relative expression levels (RNA or ribosome footprint), which show expression of the specific RF in relation to the average of all chloroplast reading frames. Translation efficiencies were calculated for each RF by subtracting the summarized log_2_-transformed signals of the total mRNA from the summarized log_2_-transformed signals of ribosome footprints. The average and standard deviation of relative abundances of ribosome footprints, total mRNA, and translation efficiency were calculated for each RF from three biological replicates. Individual data from the array-based ribosome profiling experiments are shown in Supplemental Data set S1.

### Chlorophyll-a fluorescence measurements and leaf absorptance

A F-6500 fluorometer (Jasco Inc., Groß-Umstadt, Germany) was used to measure 77K chlorophyll-a fluorescence emission spectra on freshly isolated thylakoid membranes equivalent to 10-μg chlorophyll mL^−1^. The sample was excited at 430-nm wavelength with a bandwidth of 10 nm, and the emission spectrum was recorded between 655 and 800 nm in 0.5-nm intervals with a bandwidth of 1 nm. The spectra were normalized to the PSI emission maximum at 734 nm, or to the maximum emission of PSI-LHCI in the as-mutants with shifted emission maximum.

In vivo measurements of chlorophyll-a fluorescence parameters at 22°C were performed using the modular transmittance version of the Dual-PAM (Heinz Walz GmbH). Light-response curves of linear electron flux, non-photochemical quenching (qN; Krause and Weis, 1991), and the redox state of the PSII acceptor side (qL; Kramer et al., 2004) were measured after 30 min of dark adaptation. The light intensity was increased stepwise from 0 to 2,500 μE m^−2^ s^−1^, with a measuring time of 150 s for each light intensity under light-limited conditions and of 60 s under light-saturated conditions. Linear electron transport was corrected for leaf absorptance, which was calculated from leaf transmittance and reflectance spectra as 100% minus transmittance (%) minus reflectance (%). Spectra were measured between 400- and 700-nm wavelengths using an integrating sphere attached to a photometer (V-550, Jasco Inc.). The spectral bandwidth was set to 1 nm and the scanning speed was 200 nm min^−1^.

### Thylakoid membrane isolation and photosynthetic complex quantification

Thylakoid membranes were isolated as described previously (Schöttler et al., 2004). The chlorophyll content and a/b ratio were determined in 80% (v/v) acetone according to Porra et al. (1989). The contents of PSII and the cyt b_6_f were determined from difference absorbance signals of cyt b_559_ (PSII) and the cytochromes b_6_ and f in destacked thylakoids equivalent to 50-μg chlorophyll mL^−1^ (Kirchhoff et al., 2002). All cytochromes were fully oxidized by the addition of 1 mM potassium hexacyanoferrate (III). Then, 10 mM sodium ascorbate was added to reduce the high-potential form of cyt b_559_ and cytochrome f. Finally, the addition of 10 mM sodium dithionite reduced the low potential form of cyt b_559_ and the two b-type hemes of cytochrome b_6_. Using a V-550 spectrophotometer equipped with a head-on photomultiplier (Jasco GmbH) at each of the three redox potentials, absorbance spectra were measured between 575 and 540 nm. The spectral bandwidth was 1 nm and the scanning speed 100 nm min^−1^. Ten spectra were averaged per redox condition. Difference spectra were calculated by subtracting the spectrum measured in the presence of hexacyanoferrate from the ascorbate spectrum, and by subtracting the ascorbate spectrum from the spectrum measured in the presence of dithionite, respectively. Finally, a baseline calculated between 540 and 575 nm was subtracted from the signals. Then, the difference spectra were deconvoluted using reference spectra as previously described (Kirchhoff et al., 2002). PSI was quantified from light-induced difference absorbance changes of P700. Thylakoids equivalent to 50 μg chlorophyll mL^−1^ were solubilized in the presence of 0.2% (w/v) *n*-dodecyl-β-d-maltoside (DDM). After the addition of 10 mM sodium ascorbate as the electron donor and 100 μM methylviologen as the electron acceptor, P_700_ photo-oxidation was achieved by applying a light pulse of 250 ms (2,000 μmol photons m^−2^ s^−1^). Measurements were performed with the Pc-P_700_ version of the Dual-PAM instrument (Heinz Walz GmbH). Pc contents, relative to PSI, were determined in intact leaves and then recalculated based on the absolute PSI quantification performed in isolated thylakoids (Schöttler et al., 2007).

### Protein gel electrophoresis and immunoblotting

Thylakoid proteins were separated by SDS-PAGE and then transferred to a polyvinylidene membrane (Hybond P, GE Healthcare) using a tank blot system (Perfect Blue Web M, VWR International GmbH, Darmstadt, Germany). Immunochemical detection was performed using an enhanced chemiluminescence detection reagent (ECL Prime, GE Healthcare) according to the manufacturer’s instructions. Chemiluminescence was detected on X-ray film. Antibodies against the photosynthetic proteins were purchased from Agrisera AB (Vännäs, Sweden).

## Accession numbers

Sequence data from this article can be found in the GenBank/EMBL data libraries under accession number NC_001879.2 (tobacco chloroplast genome).

## Supplemental data

**Supplemental Figure S1.** Ratios of relative average translation output and transcript accumulation levels.

**Supplemental Figure S2.** Reproducibility of transcript abundance and ribosome footprint data between biological replicates.

**Supplemental Table S1.** Summary of oligonucleotide sequences

**Supplemental Data set S1.** Data from array-based ribosome profiling experiments.

## Supplementary Material

kiaa052_Supplementary_Data

## Notes

M.A.S. and R.B. conceived the project. H.-Y.F. performed most experiments. R.G. and R.Z. performed the ribosomal footprint analysis. S.R. supervised the plant transformation and transformant regeneration. M.A.S. wrote the manuscript with input from all co-authors, and also serves as the author responsible for contact and ensures communication. All authors contributed to the editing and review of the manuscript.

The author responsible for distribution of materials integral to the findings presented in this article in accordance with the policy described in the Instructions for Authors (https://academic.oup.com/plphys) is: Mark Aurel Schöttler (schoettler@mpimp-golm.mpg.de).
