# Supplementary material for: The availability of neither D2 nor CP43 limits the biogenesis of photosystem II in tobacco
Source: Plant Physiol. 2020 Dec 9;185(3):1111–30. doi: 10.1093/plphys/kiaa052 (PMC8133689; doi:10.1093/plphys/kiaa052)
Supplement: kiaa052_Supplementary_Data [file kiaa052_supplementary_data.zip › pp.01056.2020-s01.pdf]

Supplemental Figure S1

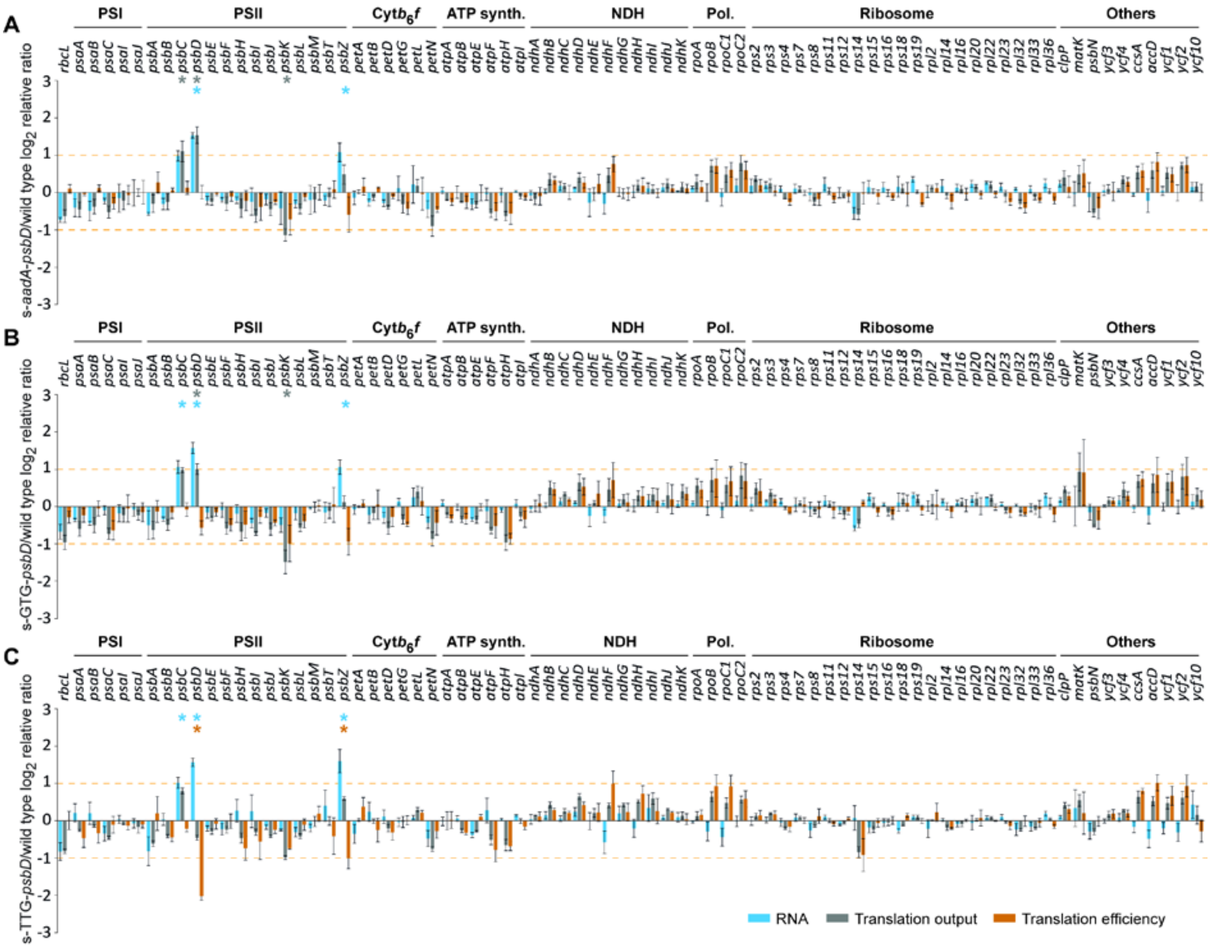

Supplemental Figure S1: Ratios of relative average translation output and transcript accumulation levels. For each gene, the ratios of relative average ribosome footprint and transcript abundances (shown in Fig. 4 and Suppl. Dataset 1) of mutants (as indicated on the y-axis) versus wild type were calculated, log_2-transformed (see Methods) and plotted. The relative average ribosome footprint and transcript abundance ratios are shown in grey and light blue for each chloroplast gene, respectively. Translation efficiencies were calculated for each gene by subtracting transcript from ribosome footprint levels (see Methods) and are plotted in orange. Column graphs represent mean values of three biological replicates (the corresponding standard deviations are shown as vertical lines). Positive and negative values indicate higher and lower relative expression levels in the mutant compared to the wild type, respectively. (A) s-*aadA*-*psbD* mutant versus wild type. (B) s-*psbD*-GTG mutant versus wild type. (C) s-*psbD*-TTG mutant versus wild type. Asterisks in the color of the respective dataset (grey, light blue, orange for ribosome footprints, RNA, translation efficiency, respectively) indicate the genes whose expression changed more than two-fold (threshold indicated by a horizontal dashed orange line). Genes are grouped in the functional categories PSI: photosystem I; PSII: photosystem II; Cyt b_6f: cytochrome b_6f complex; ATP synth.: ATP synthase; Pol.: RNA polymerase; Ribosome; NDH: NADH dehydrogenase-like complex and Others: other genes.

Supplemental Figure S2

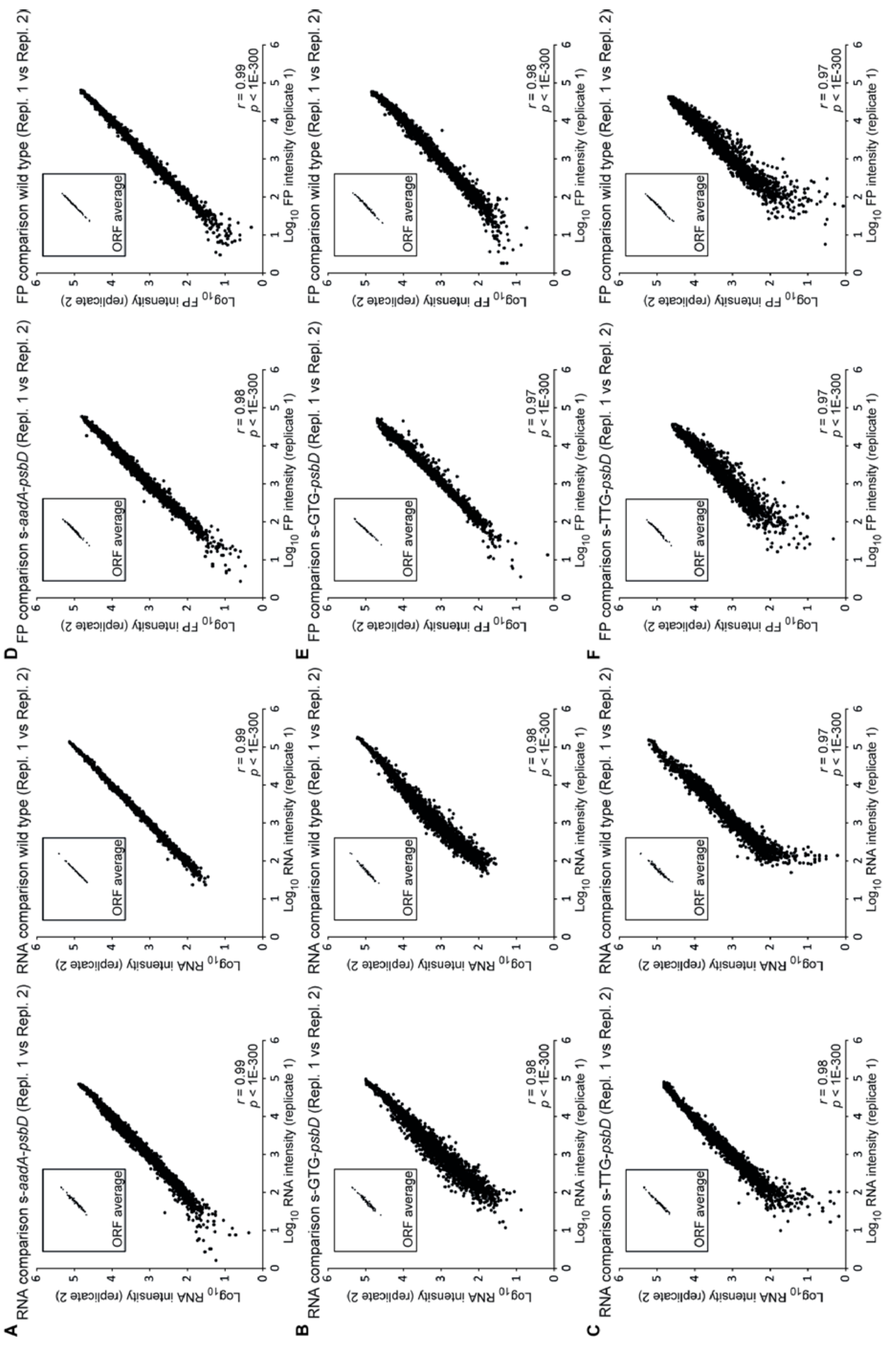

Supplemental Figure S2: Reproducibility of transcript abundance and ribosome footprint data between biological replicates. Comparisons of the log_{10}-transformed transcript (A-C, RNA) and ribosome footprint (D-F, FP, reflecting translation output) abundance values for each probe located in protein-coding regions were plotted for two representative biological replicates against each other for s-*aadA*-*psbD* mutants (uppermost panels), s-*psbD*-GTG (middle panels) and s-*psbD*-TTG (lowermost panels) and the corresponding wild types (shown next to the mutant), respectively. Pearson's r and Anova's p-values (in nEm non-superscript format for n•10m) are given within each plot. In addition, the average footprint or transcript abundances calculated for each reading frame, respectively, are compared in log_{10} scale and shown as insets. For each mutant the ribosome footprint and transcript levels were analyzed for three biological replicates (two of them are plotted here; for further comparisons see Suppl. Dataset 1).

Supplemental Table S1. Summary of oligonucleotide sequences. Restriction sites specified in the third column are underlined. The mutated nucleic acids are indicated in bold letters.

**Name**	**DNA Sequence (5'-3')**	**Restriction Site**
PsbD_F	AAAAGGTACCTGAATCATGACTATATCC	*Kpn*I
PsbD_R	AAAAGAGCTCTATGAAATGGGTTCAACG	*Sac*I
PsbD_mut1_F	TCTTCGGACGCCCCGAAAAAGATATGAGGTGCTCGGA<br>AATGGTCGAAGTAGTTGAATAGGAGGATCACT**G**TGAC	*Bsa*HI
PsbD_mut2_F	TCTTCGGACGCCCCGAAAAAGATATGAGGTGCTCGGA<br>AATGGTCGAAGTAGTTGAATAGGAGGATCACT**T**TGAC	*Bsa*HI
PsbD_mut3_F	TCTTCGGACGCCCCGAAAAAGATATGAGGTGCTCGGA<br>AATGGTCGAAGTAGTTGAATAGGA**C**GATC	*Bsa*HI
PsbD_mut4_F	TCTTCGGACGCCCCGAAAAAGATATGAGGTGCTCGGA<br>AATGGTCGAAGTAGTTGAATAGGAG**C**ATCA	*Bsa*HI
PsbD_mut5_F	TCTTCGGACGCCCCGAAAAAGATATGAGGTGCTCGGA<br>AATGGTCGAAGTAGTTGAATAGGA**CC**ATCAC	*Bsa*HI
PsbD_mut_R	GTGCTTCAGGACCCCATAGTAA	*Eco*O109I
PsbC_probe_F	ATACAACCTTGGCAGGAACG	
PsbC_probe_R	TGCCACAAATGACCTACGAA	
PsbD_probe_F	CCTAGGTCAGTTTATGGGCT	
PsbD_probe_R	GTGCTTCAGGACCCCATAGTAA	
PsbD5'_probe_F	TCGTTGCATCCGGTATTTTT	
PsbD5'_probe_R	AAGCGATCCCTTTCCTTCAT	
PsbA_probe_F	ATAGACTAGGCCAGGATCTTA	
PsbA_probe_R	ATTTTACCATGACTGCAATTT	
PetA_probe_F	ATTCTTTCCCCGGACCCTGCTA	
PetA_probe_R	CTTGCTGCCGTCGGGATAAATC	
rbcL_probe_F	TTGGCAGCATTCCGAGTAACTC	
rbcL_probe_R	TTCGCATGTACCTGCAGTAGCA	
