# Supplementary figures and images for: The availability of neither D2 nor CP43 limits the biogenesis of photosystem II in tobacco
Source: Plant Physiol. 2020 Dec 9;185(3):1111–30. doi: 10.1093/plphys/kiaa052 (PMC8133689; doi:10.1093/plphys/kiaa052)

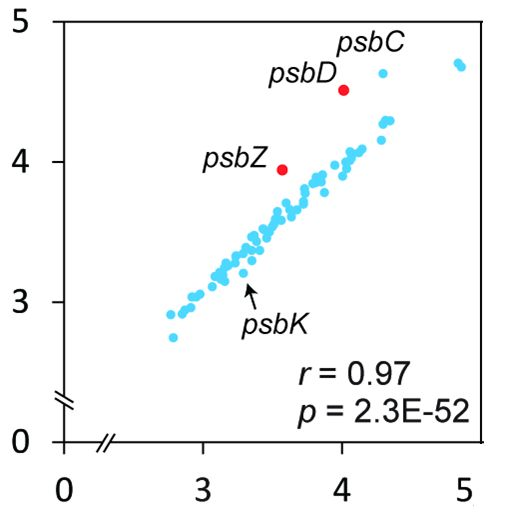

Supplement: kiaa052_Supplementary_Data [file kiaa052_supplementary_data.zip › pp.01056.2020-s03.tif]
